# The value of consciousness coaching in Parkinson’s disease: Experiences and possible impact of holistic coaching

**DOI:** 10.1016/j.prdoa.2024.100261

**Published:** 2024-06-09

**Authors:** Lousanne E.J. Tangelder, Ana L. Silva de Lima, Arjonne Laar, Nienke M. de Vries

**Affiliations:** Radboud University Medical Center, Donders Institute for Brain, Cognition and Behavior, Department of Neurology, Center of Expertise for Parkinson & Movement Disorders, Nijmegen, The Netherlands

**Keywords:** Coaching, Consciousness, Awareness, Parkinson’s disease, Disease management

## Abstract

**Background:**

People with a chronic condition such as Parkinson’s disease (PD) struggle with acceptance and finding meaning in life. Consciousness coaching could be a valuable addition in addressing these issues.

**Objective:**

We aim to evaluate the user experiences and potential effectiveness of consciousness coaching for people with PD (PwPD).

**Methods:**

We performed a pilot randomized controlled trial including PwPD in Hoehn & Yahr stage 1–3. People with cognitive impairments, severe psychiatric disorders, or those who did not have a clear issue to address with consciousness coaching, were excluded. PwPD were randomly allocated to either receiving 6 months of consciousness coaching in addition to usual care or to usual care alone. To explore experiences we performed semi-structured qualitative interviews with all PwPD in the intervention group. Potential effects were explored using questionnaires on quality of life, activities of daily life, self-management and non-motor symptoms at baseline and after 6 months.

**Results:**

We included 39 PwPD, 19 participants in the intervention group and 20 in the control group. Based on the interviews, we identified a number of themes and codes. In general PwPD experienced consciousness coaching as confronting but supportive in reaching their goals and in taking more responsibility for their lives. Quantitatively, we did not find a difference between groups for any of the outcomes.

**Conclusions:**

Consciousness coaching was considered valuable by most participants in this study and may be an interesting addition to PD treatment. We did not find any effects of the intervention on PD symptoms or quality of life.

## 1 Background

Many people struggle with problems as acceptance and meaning of life after being diagnosed with a chronic disease, such as Parkinson s disease (PD). Few people receive integrated care truly adapted to their personal needs and wishes [1], [2], [3]. Often, people with PD (PwPD) are insufficiently informed about their disease, the consequences of their disease on different aspects of their life and how psychological and spiritual matters such as acceptance and meaning of life can be affected by the disease [4], [5]. Previously, a new broad, dynamic concept of health was introduced in which health is considered not only the absence of disease, but related to the ability to adapt and to self-manage [6]. Six dimensions of health were identified that were considered equally important; one of these dimensions includes the spiritual/ existential dimension. Despite the importance of this subject for individual persons, questions related to acceptance and existence are often not addressed in regular medical care [7].

To address these issues, a specifically trained coach (a consciousness coach) could have additional value to more traditional healthcare providers. Consciousness coaching is based on traditional coaching, but goes one step further by creating awareness of new or unknown possibilities. A coach‘s role is to use effective questioning and awareness creations to empower the patient to bring clarity and purpose back into her/his life. Awareness creations can be defined as structured information given by the coach that brings a new perspective or awareness to the participant. An awareness can involve aspects of the person him/ herself, the environment or experiences of the environment. The new awareness opens up possibilities that could not be ‘seen’ by the participant before. Consciousness coaching is based on the fundamental belief that all people have all the potential resources they need to achieve the things they desire, within them. Awakening these potentials and developing them into skills and powers is one of the goals of consciousness coaching.

Here we performed a randomized controlled pilot study aiming to explore the experiences with consciousness coaching by PwPD. Secondarily we studied the potential effectiveness of this approach as compared to PwPD not receiving consciousness coaching.

## 2 Methods

### 2.1 Study design

Between 1st of October 2019 and 1st of January 2022, we performed a pilot randomized controlled trial in the region of Veldhoven, The Netherlands, comparing PwPD who received consciousness coaching in addition to usual care (intervention group) with PwPD receiving usual care alone (control group). This study was conducted in compliance with the Ethical Principles for Medical Research Involving Human Subjects, as defined in the Declaration of Helsinki. The study protocol and communication materials were approved by the local ethics committee (NL: CMO Arnhem-Nijmegen; NL72105.091.19.) All participants signed an informed consent prior to participation and data collection.

### 2.2 Participants & procedures

PwPD were recruited by physiotherapists specialized in PD who are part of the national ParkinsonNet network consisting of specialized healthcare providers [8], [9] in the region of Veldhoven. PwPD who reside in this region and had a Hoehn & Yahr stage ≤3 were eligible to participate in this study. We excluded people who had a score of <24 in the Mini-Mental State Examination, those with severe psychiatric disorders (as estimated by the treating neurologist), or those who did not have a clear issue to address with consciousness coaching (as estimated by a researcher with experience in consciousness coaching). Due to the explorative nature of this pilot study, no sample size calculation was performed. For pragmatic reasons (i.e. availability of participants and consciousness coaches), we decided to include 40 participants.

Potential participants were approached by their treating physiotherapist. They were informed about the aim of the study and about the concept of consciousness coaching. When interested, an information letter was provided and the researcher contacted the participant to provide additional information and answer questions. When the participant agreed to participate and met the inclusion criteria, we send out a set of baseline questionnaires (see outcomes). After the baseline questionnaires were filled out, randomization was performed by one of the researchers not involved in either the intervention or data collection using the Electronic Data Capture software Castor. Participants were randomly allocated to one of two groups: 1) receiving consciousness coaching in addition to usual care or; 2) to usual care alone. After 6 months, all participants received the same set of questionnaires and all participants in the intervention group participated in a semi-structured qualitative interview (interview guide, see Appendix 1). The interview was taken by an independent researcher who was not involved in the intervention in any way.

### 2.3 Intervention − consciousness coaching

Consciousness coaching consisted of 12 coaching sessions of up to 60 min, every 2 weeks. The sessions took place at the participants' homes or via video consultation. Each session made use of a standardized protocol for consciousness coaching. The first part of each session is dedicated to getting present in the moment, remembering the goals of the last session, anything else that needs attention and setting a goal for the present session. Next, the goals for the present sessions are discussed in detail and the participant becomes aware of answers that are already present unconsciously and is encouraged to talk about them. Finally, the coaching session builds a bridge to the next coaching session by creating home assignments. The home assignments are chosen by the participant, supported by the coach. In the next session, the consciousness coach asks the participant about the execution of these assignments [10], [11].

Coaching was performed by qualified and highly experienced coaches (n = 4) registered at the International Coaching Federation (https://www.coachingfederation.org). A consciousness coach has followed a 5 months training and has at least 100 h of coaching experience. It is important to mention that for this pilot we chose to work with experienced coaches in order to prevent newly trained coaches from gaining their first experience during the pilot. We provided the coaches with additional training in PD. This training consisted of a 4 h course provided by a neurologist, a PD nurse specialist, an occupational therapist and a physiotherapist, all specialized in PD. The topics that were covered included: PD motor symptoms, PD non-motor symptoms (including communication, speech, motivation and apathy), challenges in daily life, progression of the disease, treatment options and impact of medication. All participants were allocated to a personal coach based on the traveling distance between the participant and coach (in order to make it possible to have face to face sessions).

Participants in the control group received usual care. There were no restrictions considering the type and frequency of other care. Since consciousness coaching is, at this moment, not offered to PwPD, the chance of people in the control group receiving (consciousness) coaching is minimal.

### 2.4 Outcomes & data analysis

#### 2.4.1 Primary endpoint – experiences

The primary aim of this study was evaluating feasibility of consciousness coaching by mapping the experiences of PwPD using qualitative interviews. Questions in the interviews related to the experience with and perceived value of consciousness coaching. In addition, attention was paid to general positive and negative aspects of consciousness coaching, organizational aspects and factors that influence future implementation. The interviews were recorded by a voice recorder and then transcribed and processed using the software of AtlasTi (Scientific Software Development GmbH, Berlin, Germany). We implemented a framework method with deductive and inductive forms to analyze transcripts of the semi structured interviews. This means we both analyzed the data using the themes of the questionnaire as a starting point (deductive analysis) as well as an open approach in which we coded the data without any assumptions (inductive analysis). The interview data were analyzed qualitatively by two independent researchers and any disagreements on themes or codes was discussed until agreement was reached.

#### 2.4.2 Secondary endpoint – preliminary effects

Second, we studied the potential effectiveness of consciousness coaching. In this context, we used the following questionnaires: quality of life (PDQ-39) [12]; disease symptoms (MDS-UPDRS I and II) [13]; anxiety and depression (Hamilton Anxiety and Depression Scale) [14]; autonomic function (SCOPA-AUT) [15], and self-management (Patient Activation Measure- PAM) [16]. The quantitative data were analyzed using SPSS version 25. Primarily, we compared the intervention group with the control group at T1 (after 6 months) on all outcomes using a Mann-Whitney test. We also examined the within group change in both groups. Here a Wilcoxon test was performed. A p value equal or lower than 0.05 was regarded as significant.

## 3 Results

In total, 39 participants were included out of 45 potentially eligible participants. 19 participants were randomized to the intervention group and 20 to the control group. Groups had similar distribution of demographic and disease characteristics, except for age. The participants in the intervention group were significantly younger than those in the control group (Table 1).

**Table 1:** Participants characteristics (n = 39).

	**Intervention (n = 19)**	**Control (n = 20)**	**p-value**
**Age (mean ± sd)**	63 **±** 9,7 years	70 **±** 7,9 years	0.028^3^
**Sex (% men)**	58 % (n = 11)	60 % (n = 12)	0.576^1^
**Disease duration (mean ± sd)**	13,2 **±** 8,4 years	9,3 **±** 4,4 years	0.166
**Time since diagnose (mean ± sd)**	8,6 **±** 6,7 years	7,6 **±** 4,3 years	0.835
**Years of education(mean ± sd)**	19,2 **±** 6,1 years	17,9 **±** 4,9 years	0.647
**Relationship status (%)**	Married 68 % (n = 13) In a stable relationship 21 % (n = 4) Divorced 11 % (n = 2)	Married 80 %(n = 16) In a stable relationship 15 % (n = 3) Widowed 5 % (n = 1)	0.330^2^
**Employment (% working)**	16 %(n = 3)	10 %(n = 2)	0.475^1^
**Living situation**	With a partner 63 % (n = 12) Alone 5 % (n = 1) Others: 32 % (n = 6)	With a partner 90 % (n = 18) Alone 5 % (n = 1) With my children 5 % (n = 1)	0.159^2^
**Quality of Life (PDQ-39) Median (Range)**	26.59 (48.28)	24.79 (38.33)	.071^3^
**Disease severity (MDS-UPDRS I and II)** **Median (Range)**	21.00 (35.00)	24.00 (34.00)	.394^3^
**Anxiety and depression (HADS)** **Median (Range)**	11.50 (20.00)	9.00 (18.00)	.625^3^
**Self-management (PAM) Median (Range)**	77.20 (61.20)	80.60 (70.00)	.866^3^
**Autonomic dysfunction (SCOPA-AUT) Median (Range)**	20.50 (39.00)	25.00 (28.00)	.067^3^

1-Fisher’s exact test. 2-Persons Chi-square. 3-Independent Samples – Mann-Whitney *U* test.

Of the 19 people in the intervention group, 5 participants received the maximum number of 12 coaching sessions and 11 had less than 12 sessions (average of 7.9 **±** 3 sessions). These 11 participants had less sessions because their coaching question had already been answered and they felt they no longer needed coaching. Additionally, three out of the 19 participants stopped after the first session. The reason for dropping out were: a discrepancy in expectations of coaching (n = 1); too confronting (n = 1); expecting to receive tips on improving cognition (n = 1). From the 19 participants in the intervention group, 18 participated in an interview.

### 3.1 Experiences of the participants

An overview of all themes emerging from the interviews is presented in Table 2. In general, coaching was experienced as positive and valuable by most of the participants in several ways. Participants experienced it as confronting but also pleasant; as if a “mirror was held up to them”. The fact that the coach listened carefully and asked questions was experienced as very pleasant. In this way, the participant and coach build a confidential and trustworthy relation. The holistic view of the coach made the participant feel that they were seen as a person and human being instead of as a PwPD. However, coaching was also considered confronting and sometimes painful. Answering questions so openly and honestly with a coach they had never spoken to before was experienced as exciting.

**Table 2:** Qualitative results – experiences with consciousness coaching.

**Themes**	**Categories**	**Summary**
Reasons for coaching	−External −Handling emotions −Communication problems −Relationships −Sparring partner	For some of the participants the external reason for coaching was wanting to contribute to research. Others indicated that coaching was recommended by family and friends. An important reason for coaching was handling emotional topics such as restlessness and fear of the future, having the feeling of losing control, loneliness, feelings of insecurity, showing vulnerability, denial of PD related problems and acceptance. Other Reasons were related to communication like expressing needs to others, communicating about experiencing problems, not taking initiative or asking for support. The reasons for coaching related to relationships were about managing family relationships, missing support or lack of disease insights of people in the environment and managing work relations. Some participants were looking for a sparring partner in the coach; they were looking for someone who could help them reflect, get motivated to initiate action or just to have a ‘partner’ to discuss personal issues with, without straining family or friends. *“It is very confronting, but I think it also helps a lot in your acceptance and your sense of consciousness.”*
Additional value of coaching compared to other professionals	−Topics −Time −Competence	Most of the participants compared coaching to treatment by the PD nurse or psychologist (94,4%), because of similarities in topics that can be discussed with those healthcare professionals. The length and frequency of the coaching sessions were, however, much longer. There was more time to discuss emotional topics in depth and to develop a relationship based on trust. This connection was compared to the relationship with a physiotherapist and a social worker. Coaching was considered to have a more holistic view considering the participants’ needs compared to other (allied) health professionals. What differentiated most between the coach and other allied health professionals was that the coach asked the participant to take responsibility and hold them accountable for their actions more than other professionals. Also goal setting and creating awareness around limiting believes were considered different as compared to other health professionals. Some participants felt seen as an equal not just as someone with PD. *“I did not miss the fact that the coach was not a 'specialist in Parkinson's'. It was nice that it wasn't always about the disease. The medical professionals always say; “oh you have Parkinson's so you can't help it”. The coach focused more on performance and on dealing with yourself: “what do I want, what can I do, what do I do”*
Effects of coaching	−Behavioral change −Transformational Insights −Negative effects	88,8% of the participants indicated that they gained insights and perspectives that may transform their life after coaching. For example: experiencing more freedom in making decisions and having more influence on their own lives. Participants also indicated to feel more balanced, experience more self-love, less guilt, and seeing the future in a more positive way. 83 % of the participants even changed behavior: they felt more motivated to take initiatives and learned to prioritize personal needs and actions, thereby taking space for themselves and asking for help. Furthermore they processed grieve and acceptance in a conscious way. Some participants experienced negative effects because they were confronted with their own behavior, e.g. lack of taking responsibility or closed attitude. Other painful experiences were about feeling the feelings that were suppressed for a long time. Some participants also felt disappointment that the coach did not give Parkinson related solutions. *“yes, I prefer to love myself more. I would like to look for solutions more creatively. Usually I'm like oh that's not possible and that's not possible. But now it's like well okay how are we going to do it. Approach things a little more positively. Yes, I just became a happier person. Yes. It really brought me a lot.”*
Good coaching	−Building fundament −Building relationship −Effective communication −Create learning and results −Experienced −Conscious about supporting believes	Building a fundament was perceived positive and was impacted by monitoring and explaining the coaching process, −protocol and providing insights in relational behavior. A number of subjects were mentioned as being important related to building a relationship and important for effective communication: responding to the needs of the participant (83,3%), empathizing with the participant (61,1%), feeling a connection with the coach (55,5%), listening well and keeping professional distance. Some participants experienced hard times understanding the ‘coach language’ due to creating awareness and reflection. Learning and reaching results were influenced by support in setting goals, by keeping the participant accountable (55,5%), by motivating the participant (55,5%) and creating awareness around limiting believes. *“The coach can have a very focused conversation one-on-one and see what needs work. Because it is difficult for me to do that myself… you don't get to it or you just don't do it. It doesn't occur to you. I often think of it as it is”* *“And then a coach is good, who makes agreements with you that you have to keep, right?”*
Considerations for future implementation and future research on consciousness coaching	−Points for improvement −Recommendation −Reimbursement	Participants supported the idea to implement coaching in regular PD healthcare. However, reimbursement from the health insurance is an issue. Participants think that when they have to pay for coaching themselves they would not go. A referral for consciousness coaching from a general practitioner or neurologist would support the participant to get coaching. But these health professionals are not yet acquainted with coaching so gaining awareness on the potential impact of coaching should first be considered. Participants indicated that group coaching or a follow-up sessions after the first coaching cycle may be valuable additions. Some considerations for the future were: provide coaching for caregivers, make more people aware of consciousness coaching, make it accessible and provide coaching for longer than six months. In addition, the cost-effectiveness of consciousness coaching should be studied.

Many participants indicated that coaching gave them confidence that they could take more control of their own lives. Also, many participants indicated that they were more capable of self-love after coaching. Participants became more gentle with themselves and generally had a more positive perspective on life. Some of the participants were able to let go of feelings of guilt and fear about the future. Participants indicated to take more responsibility for their lives, initiate actions and communicate better about their needs.

Of the 19 participants who started the coaching series, 3 people experienced no added value from coaching. These participants stopped after the first session because they had different expectations, found it too confronting or were not open to self-reflection.

### 3.2 Preliminary effects

We found no significant differences on any of the outcomes neither between nor within groups (Table 3). We also did not find a trend towards effectiveness on any of the outcomes.

**Table 3:** Quantitative results – between and within group effects.

	**Intervention (Baseline) (n = 19)**	**Intervention (Follow-up) (n = 19)**	**p-value** **(within group)^1^**	**Control (Baseline) (n = 20)**	**Control** **(Follow-up) (n = 20)**	**p-value** **(between groups)^1^**	**p-value (within group)^1^**
**Quality of Life (PDQ-39) Median (Range)**	26.59 (48.28)	31.98 (47.55)	0.0597	24.79 (38.33)	28.23 (48.28)	0.422	0.470
**Disease severity (MDS-UPDRS I and II) Median (Range)**	21.00 (35.00)	23.50 (33.00)	0.595	24.00 (34.00)	22.00 (34.00)	0.707	0.773
**Anxiety and depression (HADS) Median (Range)**	11.50 (20.00)	12.50 (24.00)	0.695	9.00 (18.00)	11.00 (16.00)	0.635	0.708
**Self-management (PAM) Median (Range)**	77.20 (61.20)	74.60 (69.00)	0.772	80.60 (70.00)	69.40 (59.00)	0.707	0.418
**Autonomic dysfunction (SCOPA-AUT) Median (Range)**	20.50 (39.00)	26.50 (33.00)	0.266	25.00 (28.00)	25.00 (31.00)	0.987	0.977

1-Samples Mann-Whitney U Test.

Score 0–100 a higher score means worse the quality of life (PDQ-39).

score 0–272 a higher score means worse disability (MDS-UPDRS I and II),

score 0–21 a higher score means more anxiety and depression (HADS).

score 0–100 a higher score indicating higher patient activation (PAM).

score 0–10 a higher score means worse autonomic dysfunction (SCOPA-AUT).

## 4 Discussion

Here, we studied a new non-pharmacological intervention for PwPD that offers guidance beyond the disease, aiming to find ways to deal with everyday problems, emotions and to take responsibility for creating a life one desires, despite being diagnosed with a degenerative disease. Overall the participants in this study appreciated the holistic approach of the consciousness coach and they felt supported in taking actions on aspects of their lives that mattered to them. In this sense, consciousness coaching may fill a gap that exists in current healthcare where the disease instead of the person is the main focus of attention. At this moment consciousness coaching is still in it’s infancy. There are around 120 consciousness coaches in the Netherlands and 500 worldwide. Therefore, availability of adequately educated coaches may be a limitation for future implementation. In addition, consciousness coaches mainly work with a healthy population and this type of coaching has not been integrated in any form of care for people with a chronic disease. This study shows that consciousness coaching may be a valuable addition to the treatment options for people with a chronic disease such as PD.

During the interviews, it became clear that participants experienced increased self-love, more personal freedom and they learned to prioritize their needs. By becoming aware of aspects such as taking responsibility for your life, or choosing a new perspective on life, these effects can be reached. The concept that having a chronic condition like PD can also bring something positive to a person’s life and that is creates awareness of what is really important in life has been referred to as silver lining [17]. Consciousness coaching supports this awareness process.

Despite the positive appraisal by participants, we were not able to show any significant effects of the intervention on quality of life, disease symptoms, anxiety and depression, autonomic disfunction or self-management. This may have several reasons. First, the COVID-19 pandemic most probably impacted our results. Coaching, which normally takes place during in person meetings, was performed remotely for most of the sessions. This could have influenced the connection between the coach and participant and hamper true in depth discussions. On the other hand, remote coaching enabled us to proceed with this intervention during lockdown, which is also a strength of the present approach. More importantly, COVID-19 impacted the lives of PwPD hugely. One of the consequences of COVID-19 was that people with PD experienced more fear, which in turn worsened their PD symptoms [18]. In addition, social isolation also negatively impacted functioning in daily life, mood, physical activity and quality of life [19], [20], which may have interfered with the effects of consciousness coaching. Finally, the outcomes used in our study are quite general and may not be able to capture the specific effects of consciousness coaching. The effects that participants indicated in the interviews (i.e. more self love, prioritizing their needs ect.) are generally not included in standard PD questionnaires. Future studies should consider including other outcomes such as “The Silver Lining Questionnaire” which gives insight five relevant domains: relationships, appreciation of life, impact on others, inner strength, and changes in life philosophy [17]. Also, questionnaires on satisfaction with life, such as the Satisfaction with Life Scale 6 (SLS-6) may be appropriate [21]. These types of scales are probably more adequate to measure the effects of consciousness coaching than the usual PD specific questionnaires we used here. Another option that may show better results is including happiness as an outcome measure [22].

Another reason for not finding quantitative effects may be selection bias. The participants in this study were recruited by physiotherapists and subsequently screened by a researcher with experience in consciousness coaching. By only including participants that receive treatment by a physiotherapist we may have introduced selection bias to our sample. All of our participants had motor problems needing physiotherapy attention and we may, for example, have missed participants in the early stages (with very limited motor problems) that need help with accepting the disease. Future studies should use broader recruitment strategies. Despite the experience of the researcher with consciousness coaching, we still included three participants who had inappropriate expectations from coaching and who stopped the intervention after one session. Working with a standardized list of issues or subjects that are appropriate for consciousness coaching may be a way to improve the selection process of participants.

Even though we did not find a quantitative effect of consciousness coaching, the qualitative results are promising. Consciousness coaching could be a valuable addition to current healthcare for PwPD. While many different healthcare professionals are already involved [23], they are merely focused on treating disease symptoms and improving daily life functioning. How to cope with a degenerative disease and how to keep giving meaning to live is not one of the primary aims. However, current healthcare increasingly requires to focus not only on the disease, but also on the person and his/ her mental and spiritual needs.^5^ In addition, coaching is in general not one of the core competencies of many healthcare providers making it difficult for them to offer coaching as an additional service. A qualified and competent coach may give PwPD the tools to feel better and to give meaning to life, despite increasing physical and mental challenges. The qualitative results of our study supports this.

This study is not without limitations. First, the study was not powered to find statistically significant results. Instead we performed an exploratory trial in a convenience sample. Second, while we did include randomization and a control group, we were unable to blind participants for treatment allocation. Third, both placebo and nocebo effects could have been involved. A placebo effect in the intervention group based on the attention given to this group could have enlarged the actual intervention effect. Moreover, the control group might have performed worse because they were not allocated to an active intervention, which is known as the nocebo effect. Since we did not find any significant quantitative between or within group effects, we assume that the impact of the placebo and nocebo effect are minimal. Finally, the study was, as mentioned before, performed in times of the COVID pandemic which may have impacted the results. Future studies using another set of outcomes are warranted to further study potential effects, especially on the long term.

## 5 Funding sources

This work was supported by a research grant of Stichting Parkinson Nederland.

Lousanne E.J. Tangelder was supported by a research grant of Stichting Parkinson Nederland.

Ana Lígia Silva de Lima: the author has nothing to disclose.

Arjonne Laar: the author has nothing to disclose.

Nienke M de Vries was supported by a research grant from Stichting Parkinson Nederland.

## CRediT authorship contribution statement

**Lousanne E.J. Tangelder:** Writing – review & editing, Writing – original draft, Project administration, Methodology, Formal analysis, Data curation, Conceptualization. **Ana L. Silva de Lima:** Writing – review & editing. **Arjonne Laar:** Writing – review & editing, Formal analysis. **Nienke M. de Vries:** Conceptualization.

## Declaration of competing interest

Nienke M. de Vries received research grants from The Netherlands Organisation for Health Research and Development, Verily Life Sciences and the Michael J Fox Foundation. The authors declare that they have no known competing financial interests or personal relationships that could have appeared to influence the work reported in this paper.
