# Supplementary material for: The value of consciousness coaching in Parkinson’s disease: Experiences and possible impact of holistic coaching
Source: Clin Park Relat Disord. 2024 Jun 9;10:100261. doi: 10.1016/j.prdoa.2024.100261 (PMC11215329; doi:10.1016/j.prdoa.2024.100261)
Supplement: Supplementary Data 1 [file mmc1.docx]

Appendix 1

Interview Guide:

**Research question: How do People with PD experience consciousness coaching at home?**

(Our main study endpoint is feasibility/ acceptability. We will perform qualitative interviews after the intervention period.

Initial questions:

**The first 2 questions are about the process before the coaching actually started:**

1. For what reason did you participate in this study?

2. What was your expectation of a coach before the study started?

**The following questions are about the perceived effect of the coaching on you.**

1. What has been the influence or impact of coaching in your life during the coaching process?

2. What has the coaching brought you?

4. How did you experience the coaching process considering frequency, time, duration, type of contact?

5. What insights did the coaching give you?

**There are a number of different healthcare professionals involved in PD care. How does the coach relate to them:**

1. What makes you see a PD nurse?

2. What makes you see a PD psychologist?

3. What makes you go to a social worker?

4. What makes you go to a coach?

5. How do you see the role of a coach compared to these or other healthcare professionals?

**The following questions are focused on the future, would you like to think along?**

1. What does a consciousness coach add to Parkinson's care?

2. Why do you think people with Parkinson's should get coaching?

3. Wuld you like to be referred to a coach?

2. Coaching is now not covered by the insurance. Do you think it should be reimbursed?

3. Are there any questions or things that you would like to mention?
